# Trail Overexpression Inversely Correlates with Histological Differentiation in Intestinal-Type Sinonasal Adenocarcinoma

**DOI:** 10.1155/2013/203873

**Published:** 2013-10-07

**Authors:** M. Re, A. Santarelli, M. Mascitti, F. Bambini, L. Lo Muzio, A. Zizzi, C. Rubini

**Affiliations:** ^1^Department of Otorhinolaryngology, Marche Polytechnic University, 60121 Ancona, Italy; ^2^Department of Clinical Specialistic and Dental Sciences, Marche Polytechnic University, 60121 Ancona, Italy; ^3^Department of Sperimental and Clinical Medicine, University of Foggia, 71121 Foggia, Italy; ^4^Department of Neurosciences, Marche Polytechnic University, 60121 Ancona, Italy

## Abstract

*Introduction*. Despite their histological resemblance to colorectal adenocarcinoma, there is some information about the molecular events involved in the pathogenesis of intestinal-type sinonasal adenocarcinomas (ITACs). To evaluate the possible role of TNF-related apoptosis-inducing ligand (TRAIL) gene defects in ITAC, by investigating the immunohistochemical expression of TRAIL gene product in a group of ethmoidal ITACs associated with occupational exposure. *Material and Methods*. Retrospective study on 23 patients with pathological diagnosis of primary ethmoidal ITAC. Representative formalin-fixed, paraffin-embedded block from each case was selected for immunohistochemical studies using the antibody against TRAIL. Clinicopathological data were also correlated with the staining results. *Results*. The immunohistochemical examination demonstrated that poorly differentiated cases showed a higher percentage of TRAIL expressing cells compared to well-differentiated cases. No correlation was found with other clinicopathological parameters, including T, stage and relapses. *Conclusion*. The relationship between upregulation of TRAIL and poorly differentiated ethmoidal adenocarcinomas suggests that the mutation of this gene, in combination with additional genetic events, could play a role in the pathogenesis of ITAC.

## 1. Introduction

Malignant tumors of the nasal cavity and paranasal sinuses account for 0.2% of all human primary malignant neoplasms, with an incidence of 0.1–1.4 new cases/year/100,000 inhabitants [1–3].

Adenocarcinomas account for 10–20% of all primary malignant neoplasms of the sinonasal tract [4, 5]. Many of these have salivary gland origin, while others have histologic patterns resembling those of colon adenocarcinoma. This second type of sinonasal adenocarcinoma has been named intestinal-type adenocarcinoma (ITAC) and is responsible for less than 4% of the total malignancies of this region [6].

ITACs of the nasal cavity and paranasal sinuses can occur sporadically or are associated with occupational exposure to hardwood and leather dusts [7]. Exposure to wood and leather dusts increases the risk of adenocarcinoma by 500-fold [8, 9]. Findings from several studies have suggested clinical differences between ITAC arising in individuals with occupational dust exposure and ITAC arising sporadically. In fact tumors related to occupational exposure affect men in 85–90% of cases, showing a strong tendency to arise in the ethmoid sinuses [10–12].

ITACs are aggressive tumors characterized by frequent local recurrences, low incidence of distant metastases, and an overall mortality of approximately 53% [12]. Histopathological grading appears to be a significant prognostic indicator [11–13]. Surgery is considered the standard treatment.

ITAC seems to be preceded by intestinal metaplasia of the respiratory mucosa, induced by hardwood dust, leather dust, and other unknown agents, which is accompanied by a switch to an intestinal phenotype. The molecular mechanisms involved in metaplastic transformation of terminally differentiated epithelium to a phenotypically different epithelium are largely unknown.

The morphological appearance of these tumors is variable, and they may resemble conventional colorectal adenocarcinoma. The similarities between ITAC and intestinal adenocarcinoma involve their ultrastructural and immunohistochemical aspects [14–17].

Numerous studies have shown that mutations of the K-ras and TP53 genes are common in colorectal adenocarcinomas [18, 19]. In comparison, there is very little information about the molecular events involved in the pathogenesis of ITAC [16, 19–26], in contrast to even more increasing information about the molecular mechanisms involved in the pathogenesis of head and neck squamous cell carcinomas (HNSCC) [27–32].

Tumor necrosis factor (TNF)-related apoptosis-inducing ligand (TRAIL) is a TNF-family member, found in a variety of tissues [33] and with a conditional expression in several immune effector cells [34, 35]. To date, five different receptors have been identified to interact with TRAIL: TRAILR1 (DR4), TRAIL-R2 (DR5), TRAIL-R3 (DcR1), TRAIL-R4 (DcR2), and osteoprotegerin. DR4 and DR5 are two different death domain-containing membrane receptors, whereas DcR1 and DcR2 are two decoy receptors that compete for TRAIL binding with DR4 and DR5 [36–39]. The final effect of the TRAIL signaling is the induction of apoptosis by the intrinsic death pathway, recruiting the inactive form of caspase-8 [40]. Expression of TRAIL and its receptors has been detected in various human tumors, suggesting that TRAIL signaling pathway is involved in endogenous tumor surveillance [40], but the mechanism of how TRAIL and its receptors contribute to carcinogenesis remains unknown.

To gain further insight into the phenotype and possible mechanisms of ethmoidal ITAC, we investigated the expression of TRAIL, correlating with clinicopathological data.

## 2. Materials and Methods

### 2.1. Patients Selection

The samples of 23 primary ethmoidal intestinal-type adenocarcinomas were retrospectively retrieved from the archives of the Institute of Pathology, Marche Polytechnic University, Ancona, Italy. All the tumors in which the diagnosis of ITAC was confirmed were subsequently subtyped, as papillary, colonic, solid, mixed, or mucinous types, as described by Barnes [12].

The medical records of these patients were reviewed. Inclusion criteria were complete clinical data, uniformity of histological differentiation throughout the tumor sample, and the availability of sufficient material from the primary tumor for investigations. Patients with previous or synchronous second malignancies or with previous radiation therapy or chemotherapy were excluded from the study. The follow-up time ranged from 2 to 10 years (mean 4.8 years).

### 2.2. Immunohistochemistry

Four-micrometer serial sections from formalin-fixed, paraffin-embedded blocks of tumour representative areas were cut for each case. Immunohistochemical staining for TRAIL was performed using the following antigen retrieval system. Sections were deparaffinized in two changes of xylene for 10 minutes each, then, were rehydrated through graded alcohols, and immersed in 0,3% hydrogen peroxide in methanol for 30 minutes to block endogenous peroxidase activity. Sections were then washed in PBS. The tissue sections were placed in a microwave oven (Philips, Cooktyronic M720, 700 W) in a plastic Coplin jar filled with 10 mM sodium citrate buffer (pH 6,0) at 5-minute interval, the fluid level in the Coplin jar was removed from the microwave oven and allowed to cool. Slides were incubated overnight with a 1 : 50 dilution of the primary mouse antihuman TRAIL monoclonal antibody (Dako DO-7, Glostrup, Denmark). A biotin-streptavidin detection system was used with diaminobenzidine as the chromogen. Slides were washed twice with PBS and incubated with the linking reagent (biotinylated anti-immunoglobulins) for 15 minutes, at room temperature. After rinsing in PBS, the slides were incubated with the peroxidase-conjugated streptavidin label for 15 minutes at room temperature. The sections were again rinsed in PBS and incubated with diaminobenzidine for 10 minutes, in the dark. After chromogen development, slides were washed in two changes of water and counterstained with a 1 : 10 dilution of hematoxylin. The sections were then dehydrated, cleared in xylene, and mounted.

TRAIL expression and location were evaluated on histological section using a Leitz Orthoplan microscope equipped with a X 400 objective. The percentage of TRAIL positive cells was evaluated from a minimum of 1,000 cells in each case. Only nuclear staining of epithelial cells was observed, and the nuclei with a clear brown color, regardless of staining intensity, were regarded as TRAIL positive.

A negative control for TRAIL immunostaining was performed in all cases by omitting the primary antibody, which, in all instances, resulted in negative immunoreactivity. The positive control consisted of staining a human normal mammary gland epithelium.

### 2.3. Scoring of Preparations

Both the histological diagnoses and evaluation of the positivity for TRAIL were carried out indipendently by two of the authors (C.R. and A.Z.). Immunohistochemical labeling for TRAIL was classified as positive, when more than 5% of nuclei or cells were stained.

### 2.4. Statistical Analysis

The following pathological and clinical parameters were analyzed: tumour grade of differentiation (G1,G2,G3), tumour extension (T1,T2,T3,T4), tumor stage (I, II, III, IV), and TRAIL immunoreactivity. Differences in TRAIL immunoreactivity between the different groups were compared by the nonparametric Kruskal-Wallis test. Statistical significance was set at *P* < 0.05. All statistical analyses were performed using the SPSS statistical package (SPPS Inc., Chicago, IL).

## 3. Results

### 3.1. Clinical Findings

There were 21 males and 2 females, with a mean age of 66.3 years (range 54–77). There were 8 grade I, 8 grade II, and 7 grade III adenocarcinomas. 3 patients were in I stage, 7 patients were in II stage, 11 patients were in III stage, and 2 patients were in IV stage. Clinicopathological staging was determined by the TNM classification (UICC 2009).

All the patients had a known history of occupational exposure to hardwood dust, and intestinal-type adenocarcinoma was localized in all cases in the ethmoid region as confirmed by endoscopic and imaging (TC and/or MR) evaluation.

The main clinicopathologic features of the patients and the oncoprotein expression are summarized in Table 1.

### 3.2. Immunohistochemistry

The cases classified as ITAC showed a variable cellular appearance and were composed of a mixture of tall columnar absorptive cells, atypical stratified cylindrical cells similar to the cells seen in conventional colorectal adenocarcinoma, goblet cells, and large round to polygonal nondescriptive epithelial cells.

### 3.3. TRAIL Expression

**Figure 1:**
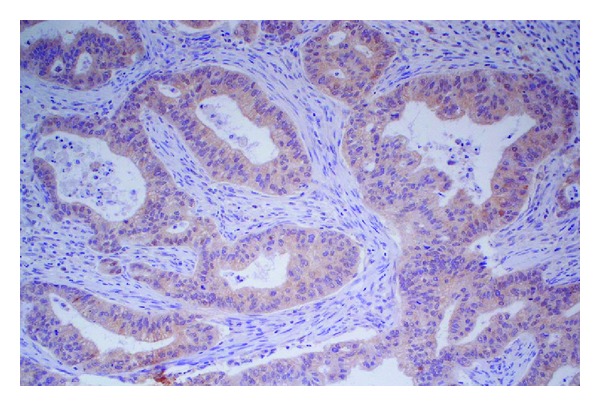
Poorly differentiated ethmoidal intestinal-type adenocarcinoma (G3) showing high percentage of TRAIL expressing cells (magnification 20x).

**Figure 2:**
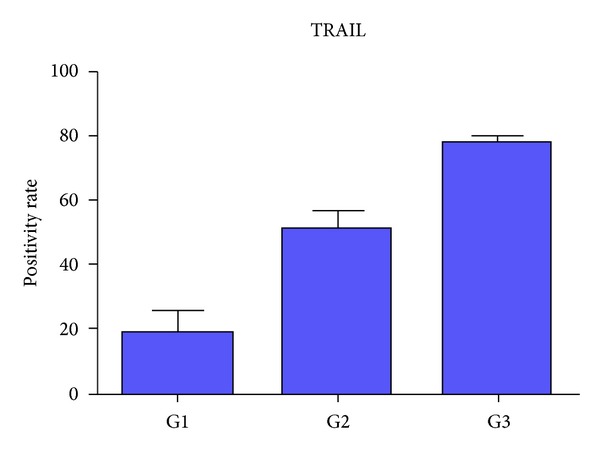
Poorly differentiated cases showed a higher percentage of TRAIL expressing cells in comparison to well-differentiated ethmoidal ITAC.

The percentage of neoplastic cells immunoreactive for TRAIL ranged from 2% to 90%. In all cases, TRAIL expression was nuclear. There was a relationship between TRAIL overexpression and the histological grading. Indeed, poorly differentiated cases (G2 and G3) showed a higher percentage of TRAIL expressing cells in comparison to well differentiated (G1) cases. No correlation between TRAIL expression and other clinicopathological parameters, including T, stage and relapses was found (Figures 1 and 2).

## 4. Discussion

Despite their histological similarity to colorectal carcinomas, there is very little information about the molecular events involved in the pathogenesis of ITAC. Several crucial pathways of tumorigenesis have been identified in colorectal adenocarcinomas [18, 19]. These pathways involve the mutation and inactivation of multiple oncogenes, tumor suppressor genes, and DNA mismatch repair genes including K-ras, APC, p53, MLH1, and MSH2 [18–20].

Working on the hypothesis that morphological similarities to colorectal adenocarcinomas might reflect equivalent genetic alterations, several authors have investigated the presence of activating mutations of Ras oncogenes and TP53 mutations in ITAC [21–25]. TP53 mutations were found in 18–44% of mostly occupational ITACs, whereas, K-Ras mutations were found in 10–15% of ITACs [21–25]. The results of these studies suggest that mutations of K-Ras and other Ras genes are relatively uncommon in ITAC, and similarly, TP53 mutations in ITACs have not been widely demonstrated.

Other studies have shown that K-Ras mutation and C-erb-2 expression could be associated with more aggressive ITACs [16, 26].

Licitra et al. found the existence of two genetic ITACs subgroups, defined by differences in TP53 mutational status or protein functionality, that strongly influence pathologic response to primary chemotherapy and, ultimately, prognosis [41].

Perez-Ordonez et al. evaluated the possible role of DNA mismatch repair (MMR) gene defects or disruptions of E-cadherin/*β*-catenin complex in ITAC by investigating the immunohistochemical expression of the MMR gene products, E-cadherin, and *β*-catenin in a group of sporadic ITACs. The preserved nuclear expression of MLH1, MSH2, MSH3, and MSH6 suggested that mutations or promoter methylation of MMR genes do not play a role in the pathogenesis of ITAC [42].

Kennedy et al. found that sinonasal ITACs have a distinctive phenotype, with all cases expressing CK20, CDX-2, and villin and most ITACs also expressing CK7, so that the expression pattern of CK7, CK20, CDX-2, and villin positive may be useful in separating these tumors from other non-ITAC adenocarcinomas of the sinonasal tract [43].

Given these findings, to explore other pathways involved in the molecular pathogenesis of ITACs, we immunohistochemically investigated the expression of the apoptosis-regulating protein TRAIL in a group of 23 ethmoidal ITAC associated with occupational exposure. This immunohistochemical expression was also retrospectively correlated with the patient outcome to evaluate their independent prognostic relevance. To the best of our knowledge, there are no previous reports on the expression of TRAIL protein in ITACs.

TRAIL is an apoptosis-inducing protein and a molecule important in inhibiting cellular immunity [44–46]. Similarly, cancer cells may use TRAIL to evade the antitumor immune response [47]. In literature, there are many works that have detected the expression of TRAIL and its receptors in several tumors, correlating this expression with clinicopathological analysis.

Trail-R1 was identified as an independent prognostic factor for disease-free survival in 128 patients with colon cancer [48]. McCarthy et al. showed that high TRAIL-R2 expression was significantly associated with decreased survival and lymph node involvement in patients with primary breast cancer [49]. Yoldas et al. revealed that TRAIL and DR4 expression were correlated with the pathological grading in patients with laryngeal squamous cell carcinoma, while the alteration in DR5 expression was correlated with the clinical staging [50]. Despite the several works, the specific mechanism of how TRAIL and its receptors contribute to carcinogenesis remains unknown.

The results of our immunohistochemical examination showed a relationship between TRAIL upregulation and the increase of the histological tumor grading. No correlation was found with other clinicopathological parameters, including T, stage and relapses.

## 5. Conclusion

Our results suggest that mutations of TRAIL expression, in combination with additional genetic events, could play a role in the pathogenesis of ITAC. However, this study showed that TRAIL expression cannot be considered as an independent prognostic factor in patients with ITACs, because there was not sufficient statistical power to detect significant associations between immunohistochemical expression of this protein and the clinicopathologic parameters of the tumors, therefore, further investigations with a larger sample are needed.

## Figures and Tables

**Table 1:** Clinicopathological data of patients and TRAIL oncoprotein expression percentage.

Patient	Age	Sex	T	Grade	CH	CHT	RT	Relapse	Trail%
1	70	M	T2	3	No	No	Yes	Yes	70
2	55	M	T3	1	Yes	Yes	Yes	No	10
3	67	M	T3	2	No	Yes	Yes	No	60
4	60	F	T1	1	Yes	No	No	No	5
5	62	M	T3	3	Yes	Yes	Yes	Yes	80
6	54	M	T3	2	Yes	No	Yes	No	35
7	54	M	T3	2	Yes	No	Yes	No	50
8	74	M	T3	1	Yes	No	Yes	No	20
9	74	M	T2	2	Yes	No	Yes	Yes	55
10	58	M	T1	1	Yes	No	Yes	No	2
11	74	M	T2	3	Yes	No	Yes	Yes	80
12	70	M	T2	2	Yes	No	Yes	No	50
13	72	M	T3	1	Yes	No	Yes	No	3
14	68	M	T3	3	Yes	No	Yes	No	70
15	77	M	T3	2	Yes	No	Yes	No	75
16	67	M	T3	1	Yes	No	Yes	No	45
17	77	M	T2	2	Yes	No	No	Yes	60
18	65	M	T3	3	Yes	No	Yes	Yes	75
19	63	M	T2	1	Yes	No	Yes	No	55
20	61	F	T1	1	Yes	No	Yes	No	5
21	65	M	T2	2	Yes	No	No	No	25
22	61	M	T4a	3	Yes	No	Yes	Yes	90
23	76	M	T4b	3	Yes	No	No	Yes	80

T: tumor extension, CH: surgery, CHT: chemotherapy, RT: radiotherapy.

## References

[1] Robin PE, Powell DJ, Stansbie JM (1979). Carcinoma of the nasal cavity and paranasal sinuses: incidence and presentation of different histological types. *Clinical Otolaryngology and Allied Sciences*.

[2] Batsakis JG, Rice DH, Solomon AR (1980). The pathology of head and neck tumors: squamous and mucous-gland carcinomas of the nasal cavity, paranasal sinuses, and larynx, part VI. *Head and Neck Surgery*.

[3] Re M, Pasquini E (2013). Nasopharyngeal mucoepidermoid carcinoma in children. *International Journal of Pediatric Otorhinolaryngology*.

[4] Weber AL, Stanton AC (1984). Malignant tumors of the paranasal sinuses: radiological, clinical, and histopathologic evaluation of 200 cases. *Head and Neck Surgery*.

[5] Iacoangeli M, Di Rienzo A, Re M (2013). Endoscopic endonasal approach for the treatment of a large clival giant cell tumor complicated by an intraoperative internal carotid artery rupture. *Cancer Management and Research*.

[6] Lopez JI, Nevado M, Eizaguirre B, Perez A (1990). Intestinal-type adenocarcinoma of the nasal cavity and paranasal sinuses. A clinicopathologic study of 6 cases. *Tumori*.

[7] Kleinsasser O, Schroeder H-G (1988). Adenocarcinomas of the inner nose after exposure to wood dust: morphological findings and relationships between histopathology and clinical behavior in 79 cases. *Archives of Oto-Rhino-Laryngology*.

[8] Leclerc A, Cortes MM, Gerin M, Luce D, Brugere J (1994). Sinonasal cancer and wood dust exposure: results from a case-control study. *American Journal of Epidemiology*.

[9] Stellman SD, Demers PA, Colin D, Boffetta P (1998). Cancer mortality and wood dust exposure among participants in the American Cancer Society Cancer Prevention Study-II (CPS-II). *American Journal of Industrial Medicine*.

[10] Hadfield EH (1970). A study of adenocarcinoma of the paranasal sinuses in woodworkers in the furniture industry. *Annals of the Royal College of Surgeons of England*.

[11] Klintenberg C, Olofsson J, Hellquist H (1984). Adenocarcinoma of the ethmoid sinuses. A review of 28 cases with special reference to wood dust exposure. *Cancer*.

[12] Barnes L (1986). Intestinal-type adenocarcinoma of the nasal cavity and paranasal sinuses. *American Journal of Surgical Pathology*.

[13] Franchi A, Gallo O, Santucci M (1999). Clinical relevance of the histological classification of sinonasal intestinal-type adenocarcinomas. *Human Pathology*.

[14] Mills SE, Fechner RE, Cantrell RW (1982). Aggressive sinonasal lesion resembling normal intestinal mucosa. *American Journal of Surgical Pathology*.

[15] Batsakis JG, Mackay B, Ordonez NG (1984). Enteric-type adenocarcinoma of the nasal cavity: an electron microscopic and immunocytochemical study. *Cancer*.

[16] McKinney CD, Mills SE, Franquemont DW (1995). Sinonasal intestinal-type adenocarcinoma: immunohistochemical profile and comparison with colonic adenocarcinoma. *Modern Pathology*.

[17] Franchi A, Massi D, Baroni G, Santucci M (2003). CDX-2 homeobox gene expression. *American Journal of Surgical Pathology*.

[18] Fearon ER, Vogelstein B (1990). A genetic model for colorectal tumorigenesis. *Cell*.

[19] Chung DC (2000). The genetic basis of colorectal cancer: insights into critical pathways of tumorigenesis. *Gastroenterology*.

[20] Calvert PM, Frucht H (2002). The genetics of colorectal cancer. *Annals of Internal Medicine*.

[21] Saber AT, Nielsen LR, Dictor M, Hagmar L, Mikoczy Z, Wallin H (1998). K-ras mutations in sinonasal adenocarcinomas in patients occupationally exposed to wood or leather dust. *Cancer Letters*.

[22] Pérez P, Dominguez O, González S, Triviño A, Suárez C (1999). ras gene mutations in ethmoid sinus adenocarcinoma: prognostic implications. *Cancer*.

[23] Wu T-T, Barnes L, Bakker A, Swalsky PA, Finkelstein SD (1996). K-ras-2 and p53 genotyping of intestinal-type adenocarcinoma of the nasal cavity and paranasal sinuses. *Modern Pathology*.

[24] Perrone F, Oggionni M, Birindelli S (2003). TP53, P14ARF, P16INK4a and H-ras gene molecular analysis in intestinal-type adenocarcinoma of the nasal cavity and paranasal sinuses. *International Journal of Cancer*.

[25] Re M, Magliulo G, Tarchini P (2011). p53 and Bcl-2 over-expression inversely correlates with histological differentiation in occupational ethmoidal intestinal-type sinonasal adenocarcinoma. *International Journal of Immunopathology and Pharmacology*.

[26] Gallo O, Franchi A, Fini-Storchi I (1998). Prognostic significance of c-erbB-2 oncoprotein expression in intestinal-type adenocarcinoma of the sinonasal tract. *Head & Neck*.

[27] Crowe DL, Hacia JG, Hsieh C-L, Sinha UK, Rice DH (2002). Molecular pathology of head and neck cancer. *Histology and Histopathology*.

[28] Lo Muzio L, Santarelli A, Caltabiano R (2005). p63 overexpression associates with poor prognosis in head and neck squamous cell carcinoma. *Human Pathology*.

[29] Re M, Magliulo G, Ferrante L (2013). p63 expression in laryngeal squamous cell carcinoma is related to tumor extension, histologic grade, lymph node involvement and clinical stage. *Journal of Biological Regulators & Homeostatic Agents*.

[30] Artico M, Bianchi E, Magliulo G (2012). Neurotrophins, their receptors and KI-67 in human GH-secreting pituitary adenomas: an immunohistochemical analysis. *International Journal of Immunopathology and Pharmacology*.

[31] Re M, Romeo R, Mallardi V (2002). Paralateral-nasal malignant schwannoma with rhabdomyoblastic differentiation (Triton tumor). Report of a case. *Acta Otorhinolaryngologica Italica*.

[32] Verdolini R, Amerio P, Goteri G (2001). Cutaneous carcinomas and preinvasive neoplastic lesions. Role of MMP-2 and MMP-9 metalloproteinases in neoplastic invasion and their relationship with proliferative activity and p53 expression. *Journal of Cutaneous Pathology*.

[33] Wiley SR, Schooley K, Smolak PJ (1995). Identification and characterization of a new member of the TNF family that induces apoptosis. *Immunity*.

[34] Zamai L, Ahmad M, Bennett IM, Azzoni L, Alnemri ES, Perussia B (1998). Natural killer (NK) cell-mediated cytotoxicity: differential use of TRAIL and Fas ligand by immature and mature primary human NK cells. *Journal of Experimental Medicine*.

[35] Griffith TS, Wiley SR, Kubin MZ, Sedger LM, Maliszewski CR, Fanger NA (1999). Monocyte-mediated tumoricidal activity via the tumor necrosis factor-related cytokine, TRAIL. *Journal of Experimental Medicine*.

[36] Wu GS (2009). TRAIL as a target in anti-cancer therapy. *Cancer Letters*.

[37] Mahalingam D, Szegezdi E, Keane M, Jong SD, Samali A (2009). TRAIL receptor signalling and modulation: are we on the right TRAIL?. *Cancer Treatment Reviews*.

[38] Ashkenazi A, Holland P, Eckhardt SG (2008). Ligand-based targeting of apoptosis in cancer: the potential of recombinant human apoptosis ligand 2/tumor necrosis factor-related apoptosis-inducing ligand (rhApo2L/TRAIL). *Journal of Clinical Oncology*.

[39] Wang S (2008). The promise of cancer therapeutics targeting the TNF-related apoptosis-inducing ligand and TRAIL receptor pathway. *Oncogene*.

[40] Kumamoto H, Ooya K (2005). Expression of tumor necrosis factor *α*, TNF-related apoptosis-inducing ligand, and their associated molecules in ameloblastomas. *Journal of Oral Pathology and Medicine*.

[41] Licitra L, Suardi S, Bossi P (2004). Prediction of TP53 status for primary cisplatin, fluorouracil, and leucovorin chemotherapy in ethmoid sinus intestinal-type adenocarcinoma. *Journal of Clinical Oncology*.

[42] Perez-Ordonez B, Huynh NN, Berean KW, Jordan RCK (2004). Expression of mismatch repair proteins, *β* catenin, and E cadherin in intestinal-type sinonasal adenocarcinoma. *Journal of Clinical Pathology*.

[43] Kennedy MT, Jordan RCK, Berean KW, Perez-Ordoñez B (2004). Expression pattern of CK7, CK20, CDX-2, and villin in intestinal-type sinonasal adenocarcinoma. *Journal of Clinical Pathology*.

[44] Sanlioglu AD, Dirice E, Elpek O (2009). High TRAIL death receptor 4 and decoy receptor 2 expression correlates with significant cell death in pancreatic ductal adenocarcinoma patients. *Pancreas*.

[45] Dirice E, Sanlioglu AD, Kahraman S (2009). Adenovirus-mediated TRAIL gene (Ad5hTRAIL) delivery into pancreatic islets prolongs normoglycemia in streptozotocin-induced diabetic rats. *Human Gene Therapy*.

[46] Cheung S-SC, Metzger DL, Wang X (2005). Tumor necrosis factor-related apoptosis-inducing ligand and CD56 expression in patients with type 1 diabetes mellitus. *Pancreas*.

[47] Trauzold A, Siegmund D, Schniewind B (2006). TRAIL promotes metastasis of human pancreatic ductal adenocarcinoma. *Oncogene*.

[48] Sträter J, Hinz U, Walczak H (2002). Expression of TRAIL and TRAIL receptors in colon carcinoma: TRAIL-R1 is an independent prognostic parameter. *Clinical Cancer Research*.

[49] McCarthy MM, Sznol M, DiVito KA, Camp RL, Rimm DL, Kluger HM (2005). Evaluating the expression and prognostic value of TRAIL-R1 and TRAIL-R2 in breast cancer. *Clinical Cancer Research*.

[50] Yoldas B, Ozer C, Ozen O (2011). Clinical significance of TRAIL and TRAIL receptors in patients with head and neck cancer. *Head & Neck*.

